# Almotriptan 12.5 mg in menstrually related migraine: A randomized, double-blind, placebo-controlled study

**DOI:** 10.1177/0333102410378048

**Published:** 2011-01

**Authors:** Gianni Allais, Gennaro Bussone, Giovanni D’Andrea, Franca Moschiano, Florindo d’Onofrio, Fabio Valguarnera, Gian Camillo Manzoni, Licia Grazzi, Rita Allais, Chiara Benedetto, Giancarlo Acuto

**Affiliations:** 1 University of Turin, Italy.; 2 Neurological Hospital Carlo Besta, Italy.; 3 Headache and Cerebrovascular Center, Italy.; 4 Mandic Hospital, Italy.; 5 Merate Hospital, Italy.; 6 Moscati Hospital, Italy.; 7 Sestri Ponente Hospital, Italy.; 8 University of Parma, Italy.; 9 Almirall SpA, Italy.

**Keywords:** Almotriptan, headache, menstrually related migraine, placebo, randomized controlled trial

## Abstract

*Background*: Menstrually related migraine (MRM) affects more than half of female migraineurs. Because such migraines are often predictable, they provide a suitable target for treatment in the mild pain phase. The present study was designed to provide prospective data on the efficacy of almotriptan for treatment of MRM.

*Methods*: Premenopausal women with MRM were randomized to almotriptan (*N* = 74) or placebo (*N* = 73), taken at onset of the first perimenstrual migraine. Patients crossed over to the other treatment for the first perimenstrual migraine of their second cycle, followed by a two-month open-label almotriptan treatment period.

*Results*: Significantly more patients were pain-free at two hours (risk ratio [RR] = 1.81; *p = *.0008), pain-free from 2–24 hours with no rescue medication (RR = 1.99; *p = *.0022), and pain-free from 2–24 hours with no rescue medication or adverse events (RR = 1.94; *p = *.0061) with almotriptan versus placebo. Nausea (*p = *.0007) and photophobia (*p = *.0083) at two hours were significantly less frequent with almotriptan. Almotriptan efficacy was consistent between three attacks, with 56.2% of patients pain-free at two hours at least twice. Adverse events were similar with almotriptan and placebo.

*Conclusion*: Almotriptan was significantly more effective than placebo in women with MRM attacks, with consistent efficacy in longer-term follow-up.

## Introduction

Menstrually related migraine (MRM) affects >50% of female migraineurs, with <10% having migraines exclusively in the perimenstrual period (PMP) (1). The pathophysiology of MRM appears to involve an abnormal neurotransmitter and neurohormonal response, or abnormal release of prostaglandins triggered by the cyclical drop of estrogen levels (2,3). Neurobiological research suggests that activated central sensory neurons may gradually become sensitized, leading to progression of pain and increasing sensitivity to extracranial stimuli (4). These findings provide a pathophysiological rationale for treatment at the first sign of pain; as MRM is often predictable, it is an excellent target for strategies initiated during the mild pain phase.

Women with MRM may benefit from short-term prevention (5,6), but the condition is characterized by a relatively low attack frequency. There is, therefore, a clear need for effective acute treatment over and above the benefits of short-term preventive treatment. The selective 5-hydroxytryptamine 1B/1D receptor agonists (triptans) are generally the most effective agents available (7) and may therefore be preferable for MRM in view of its difficult-to-treat nature (1).

In a retrospective analysis of data from women with MRM from a randomized, comparative study of almotriptan versus zolmitriptan for acute treatment of migraine, almotriptan was effective and well tolerated (8), while a post-hoc analysis of the placebo-controlled AXERT Early Migraine Intervention Study (AEGIS) study showed that almotriptan was similarly effective in MRM and non-MRM (9). To provide prospective data on almotriptan in MRM, we conducted a randomized, double-blind, placebo-controlled trial.

## Methods

### Objectives

The primary objective was to demonstrate the superiority of almotriptan over placebo in the percentage of patients free of pain two hours after drug intake during an MRM attack. Secondary objectives were to investigate the efficacy and tolerability of almotriptan versus placebo in women with MRM, and the consistency of treatment with almotriptan during open-label follow-up.

### Study design

The study (EudraCT: 2005-000244-90) was a two-month, multicenter, randomized, double-blind, placebo-controlled crossover trial followed by a two-month active treatment open-label follow-up evaluation to assess consistency. The study was performed in the neurology, headache or gynecology departments at seven hospitals in Italy, in accordance with Good Clinical Practice. The protocol was approved by the ethics committees of all participating centers and written informed consent was obtained from each patient.

Patients enrolled in the study attended the clinic for five study visits, including the screening assessment. Each visit other than the screening visit took place two to six days after each migraine attack recorded in the study. At each visit patients recorded migraine pain intensity, adverse events and concomitant medications in a diary. 

Patients were randomized 1:1 to receive double-blind oral almotriptan malate or identical, matching placebo. Patients were assigned a randomization number in ascending order by the Statistics Group within Almirall SA., according to the relevant standard operating procedure. The randomization list was computer-generated, and randomization data were kept strictly confidential, accessible only by authorized persons. Data were unblinded only when the trial was completed and the data verified and locked. The success of blinding was not formally evaluated during the study.

Study medication was self-administered at the onset of the first migraine attack occurring during the PMP (defined as day −2 to +3 of the menstrual cycle). Participants took one 12.5 mg tablet, preferably in the first hour after pain onset or, if possible, when the pain was still of mild intensity, and not more than two hours after pain onset. Patients then crossed over to the other double-blind treatment (almotriptan or placebo) for the first migraine in the PMP of their second menstrual cycle. Two boxes (one box for the first menstrual migraine attack of one menstrual cycle and the other for the first menstrual migraine attack occurring in the subsequent menstrual cycle) per patient were provided, each containing two tablets of study medication or placebo in sealed blisters. One tablet was to be used for the treatment of the attack, with the other to be used only in case of recurrence. In the open-label phase, all patients received almotriptan for the first PMP migraine in their third and fourth menstrual cycles. 

### Patients

Patients were almotriptan-naive women aged 18–50 years, from any ethnic group, who experienced MRM, as defined according to the criteria of the International Headache Society (10). Patients were required to have regular menstrual cycles, with at least a one-year history of migraine and a six-month history of regularly occurring MRM (migraine attacks without aura, occurring on days −2 to +3 of menstruation in at least two of three menstrual cycles, with or without additional attacks at other times of the cycle). Participants were required to be in good general health, and women of childbearing potential were required to use an adequate form of contraception. Key exclusion criteria included: any other type of headache that would confound diagnosis of MRM; migraine headaches that did not typically have a mild pain phase, that were typically associated with vomiting, or hemiplegic or basilar migraines; chronic daily headache (≥15 headache days per month for the previous six months) or ≥6 migraines per month for the previous three months; use of more than one medication (for any reason) known to be effective in migraine prophylaxis; use of sustained-release opioids, or semisynthetic or long-acting opioids, within seven days before study entry; use of systemic or injectable corticosteroids within 30 days before study entry; previous overuse (>2 days per week) of analgesics, benzodiazepine sedative hypnotics, antiemetics, triptans, opioids or ergotamine-type medications; significant unstable medical disease or a history of a significant mental disorder; or a current or recent history, or suspected history, of substance dependence or abuse. Lactating women were also excluded from the study.

At baseline, patients were also required to provide a verifiable diary of their migraine history covering at least their preceding two menstrual cycles, their medical history in general and their latest medication history.

### Assessments

Headache pain intensity was rated by patients on a 4-point scale: (0 = no headache pain; 1 = mild headache pain, allowing normal activity; 2 = moderate headache pain, disturbing but not prohibiting normal activity, bed rest is unnecessary; 3 = severe headache pain, normal activity has to be discontinued, bed rest may be necessary). Migraine-associated symptoms (nausea, vomiting, phonophobia, photophobia, osmophobia), migraine duration and use of rescue medication were recorded throughout the study. Rescue medications included nonsteroidal anti-inflammatory drugs and paracetamol, and other agents that investigators considered to be appropriate for each patient at their center. Triptans and ergotamine-containing drugs were not permitted as rescue medication. Tolerability was assessed in terms of adverse events, physical examinations and vital signs. 

### Endpoints and analysis

According to sample size calculations, 130 evaluable patients were required to demonstrate superiority of almotriptan over placebo, and thus it was planned to screen and randomize 160 patients to account for an estimated 20% of withdrawals after randomization. A sample size of 130 patients (pairs of observations) achieves 90% power to detect an odds ratio (OR) of 2.00 using a two-sided McNemar test with a significance level of 0.05 and the proportion of patients free of pain at two hours as primary endpoint. The OR is equivalent to a difference between two paired proportions of 0.250, which occurs when the proportion of patients responding to almotriptan and not to placebo is 0.500 and the proportion of patients responding to placebo and not to almotriptan is 0.250. The proportion of discordant pairs is 0.750.

The primary endpoint was the percentage of patients free of pain at two hours after drug intake. Secondary endpoints included percentage of patients pain-free at time points from 0.25 to 24 hours after drug intake; percentage of patients being sustained pain-free (SPF; defined as pain-free from 2 to 24 hours with no rescue medication use); percentage of patients being SPF with no adverse events (SNAE); rate of recurrence (defined as onset of a new attack within 24 hours of successful drug treatment of the first migraine attack); percentage of patients with rescue medication intake; evolution of migraine-associated symptoms (percentage of patients with nausea, vomiting, phonophobia, photophobia or osmophobia 0.25–24 hours after study drug intake). 

The evaluation of primary endpoint and all the secondary endpoints, with the exception of “duration of migraine attack”, was performed by means of a generalized linear model implemented with binomial distribution, log-link function and generalized estimating equations. The model effects were treatment sequence (almotriptan–placebo or placebo–almotriptan), treatment (almotriptan or placebo) and the period (first and second). As this was a crossover study, a compound symmetry variance–covariance matrix was employed to account for clustered data (repeated measures). 

Results were reported as RRs with the associated 95% confidence interval (CI) and two-tailed *p* values. The primary efficacy population was the modified intent-to-treat (mITT) population, defined as all patients who received at least one dose of study medication and for whom data on the primary endpoint were available (i.e. double-blind phase completers). A per-protocol analysis, from which patients with major protocol violations were excluded, was also performed. The safety population included all patients who received at least one dose of study medication. A post-hoc analysis was conducted to assess the efficacy of almotriptan in comparison with placebo in the subgroups of patients with pain of moderate/severe (intensity score = 2−3) or mild (intensity score = 1) intensity at headache onset.

## Results

### Patient disposition

**Figure 1.**
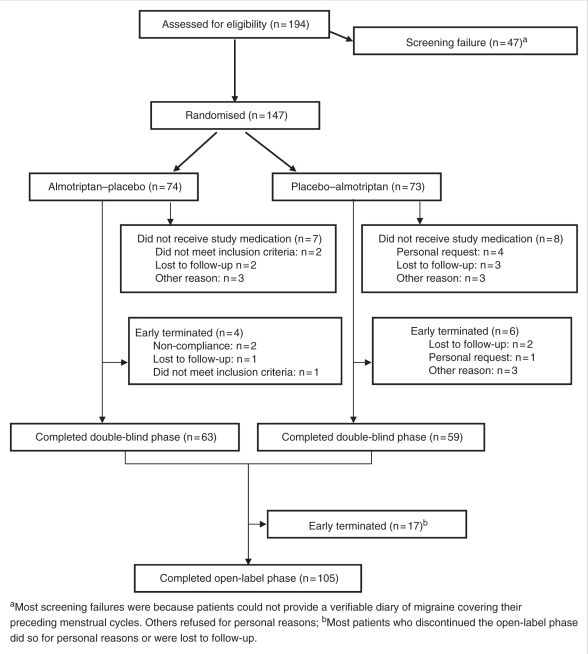
Patient disposition during the study.

The study was conducted between May 2005 and October 2008. In total, 194 patients were screened, of whom 147 were randomized to almotriptan–placebo (ALM/PLA) (*N* = 74) or placebo–almotriptan (PLA/ALM) (*N* = 73) (Figure 1). Of the 147 randomized patients, 132 received at least one dose of study medication (ALM/PLA, *N* = 67; PLA/ALM, *N* = 65) and were included in the safety analysis. Baseline characteristics were similar in the two treatment arms; overall, the mean (± standard deviation [SD]) age of patients was 34.9 ± 8.0 years, and all patients were Caucasian. The double-blind phase was completed by 63/74 (85.1%) and 59/73 (80.8%) patients in the ALM/PLA and PLA/ALM arms, respectively, and these patients formed the mITT population (*N* = 122). The most common reasons for withdrawal were loss to follow-up (*N* = 8) and personal request (*N* = 5) (Figure 1). The per-protocol population included 55 patients in each treatment arm. The post-hoc analysis included 68 patients in the mild subgroup (almotriptan *N* = 36; placebo *N* = 32) and 176 in the moderate/severe subgroup (almotriptan, *N* = 86; placebo *N* = 90).

Of the 122 patients who entered the open-label phase, 105 (86.1%) completed the study. The most common reason for withdrawal in the open-label phase was loss to follow-up.

### Efficacy — double-blind phase

**Figure 2.**
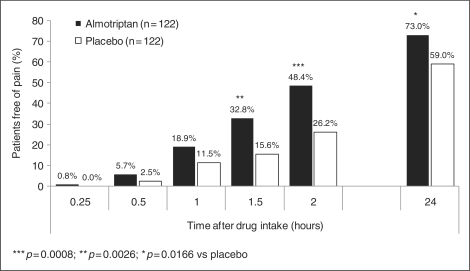
Percentage of patients pain-free at each time point after drug intake (modified intent-to-treat [mITT] population).

Almotriptan was associated with a significantly higher percentage of patients free of pain at two hours after drug intake compared with placebo (48.4% [*N* = 59/122] vs. 26.2% [*N* = 32/122]; RR, 1.81 [95% CI, 1.28–2.57]; *p = *.0008) (Table 1; Figure 2). The result was confirmed in the per-protocol population (49.1% [*N* = 54/110] vs. 23.6% [*N* = 26/110]; RR, 2.02 [95% CI, 1.37–2.99]; *p = *.0004). In the post-hoc analysis, almotriptan was associated with a significantly higher percentage of patients free of pain at two hours in the mild subgroup (69.4% vs. 21.9%; *p = *.0011) but not in the moderate/severe subgroup (39.5% vs. 27.8%; *p = *.1100) (Table 1).

**Table 1.** Percentage of patients pain-free at 2 hours, SPF and SNAE

	2 hours pain-free				SPF				SNAE			
	*N*	%	RR (95% CI)	*p* value	*N*	%	RR (95% CI)	*p *value	*N*	%	RR (95% CI)	*p* value
All patients												
Almotriptan (*N* = 122)	59	48.4	1.81 (1.28–2.57)	.0008	44	36.1	1.99 (1.28–3.09)	.0022	41	33.6	1.94 (1.21–3.13)	.0061
Placebo (*N* = 122)	32	26.2			21	17.2			20	16.4		
Mild subgroup (post-hoc analysis)												
Almotriptan (*N* = 36)	25	69.4	3.11 (1.58–6.14)	.0011	19	52.8	2.01 (0.93–4.36)	.0766	17	47.2	1.71 (0.67–4.39)	.2611
Placebo (*N* = 32)	7	21.9			7	21.9			7	21.9		
Moderate/severe subgroup (post-hoc analysis)												
Almotriptan (*N* = 86)	34	39.5	1.42 (0.92–2.18)	.1100	25	29.1	1.88 (1.08–3.30)	.0268	24	27.9	1.95 (1.07–3.57)	.0304
Placebo (*N* = 90)	25	27.8	14	15.6	13	14.4

SPF = sustained pain-free (pain-free from 2–24 hours with no rescue medication). SNAE = pain-free from 2–24 hours with no rescue medication or adverse events. CI = confidence interval; RR = risk ratio.

Overall, 36.1% of patients were classed as SPF while receiving almotriptan, compared with 17.2% while receiving placebo (*p = *.0022); the result was also statistically significant in the moderate/severe subgroup (29.1% vs. 15.6%; *p = *.0268) but failed to achieve significance in the mild subgroup (52.8% vs. 21.9%; *p = *.0766) (Table 1). Similarly, almotriptan was associated with a significantly higher percentage of patients classed as SNAE compared with placebo (33.6% vs. 16.4%; *p = *.0061) (Table 1). Again, this difference was statistically significant in the moderate/severe subgroup (27.9% vs. 14.4%; *p = *.0304) but not in the mild subgroup (47.2% vs. 21.9%; *p = *.2611).

During the double-blind phase, rescue medication was used by 39.3% of patients (*N* = 48/122) while receiving almotriptan and 59.8% (*N* = 73/122) while receiving placebo (RR, 0.65 [95% CI, 0.52–0.83]; *p = *.0004). In the mild and moderate/severe subgroups, the percentages were 33.3% (*N* = 12/36) vs. 59.4% (*N* = 19/32) (RR, 0.55 [95% CI, 0.33–0.92]; *p = *0.0227) and 41.9% (*N* = 36/86) vs. 60.0% (*N* = 54/90) (RR, 0.69 [95% CI, 0.52–0.91]; *p = *.0078), respectively. The duration of migraine attack was significantly shorter in patients receiving almotriptan compared with placebo (7.5 vs. 10.8 hours; *p = *.0170), but was not significantly different between the two treatment arms in either the mild or moderate/severe subgroups (5.6 vs. 10.0 hours; *p = *0.080 and 8.4 vs. 11.1 hours; *p = *0.093, respectively) (Table 2).

**Table 2.** Mean duration (hours) of migraine attacks during double-blind treatment

	Pain-free		
	Migraine duration (mean ± SD)	Mean treatment difference (95% CI)	*p* value
All patients			
Almotriptan (*N* = 122)	7.5 ± 10.1	−3.3 (−5.9; −0.6)	.0170
Placebo (*N* = 122)	10.8 ± 11.1		
Mild subgroup (post-hoc analysis)			
Almotriptan (*N* = 36)	5.6 ± 9.2	−4.4 (−9.3; 0.5)	.0801
Placebo (*N* = 32)	10.0 ± 11.1		
Moderate/severe subgroup (post-hoc analysis)			
Almotriptan (*N* = 86)	8.4 ± 10.4	−2.7 (−5.9; 0.5)	.0933
Placebo (*N* = 90)	11.1 ± 11.1		

SD = standard deviation. CI = confidence interval.

Migraine recurrence within 24 hours of successful treatment occurred in 28.8 % of patients (*N* = 17/59) during almotriptan treatment and 31.3% (*N* = 10/32) during placebo treatment (*p = *.9349).

The incidence of nausea at two hours after drug intake was significantly lower while patients were receiving almotriptan compared with placebo (19.0% [*N* = 23/121] vs. 36.7% [*N* = 44/120]; RR, 0.52 [95% CI, 0.36–0.76]; *p = *.0007 [Wilcoxon rank-sum test]). Similarly, the incidence of photophobia at two hours post-medication (33.1% [*N* = 40/121] vs. 49.2% [*N* = 59/120]; RR, 0.67 [95% CI, 0.50–0.90; *p = *0.0083 [Wilcoxon rank-sum test]) was significantly lower during almotriptan treatment compared with placebo. No between-group differences in vomiting, phonophobia or osmophobia at two hours after drug intake were reported. 

### Efficacy — open-label follow-up

During the open-label period, migraine data were available for the third migraine attack from 110 patients and for the fourth migraine attack from 105 patients. During the two menstrual cycles of the open-label follow-up period, the percentage of patients pain-free at two hours after medication intake in the mITT population was 55.5–59.0% (mild subgroup, 62.5–69.4%; moderate/severe subgroup, 48.6–56.9%). The percentage ranged from 0.9–1.0% at 15 minutes post-intake to 64.8–71.8% at 24 hours. The percentages of patients classed as SPF and SNAE were 40.0–41.8% and 36.2–39.1%, respectively (mild subgroup, 47.5–61.1% and 45.0–55.6%; moderate/severe subgroup, 32.4–35.4% and 30.8–31.1%). Rescue medication was used by 17.1–17.3% of patients in the mITT population, 12.5–13.9% in the mild subgroup and 18.9–20.0% in the moderate/severe subgroup. The efficacy of almotriptan in terms of percentage of patients pain-free at two hours, SPF, and SNAE was highly consistent between the three attacks recorded in the study. Overall, 59 of 105 patients (56.2%) responded to treatment in at least two of three migraine attacks (i.e. were pain-free at two hours post-intake at least twice out of three attacks), while 42 (40.0%) and 39 (37.1%) patients were SPF and SNAE, respectively, at least twice out of three cycles.

The recurrence rate in the open-label phase was 29.5–33.9%. The incidence of migraine-associated symptoms was generally numerically lower in the open-label phase than in the double-blind phase. At two hours after intake, the incidences were nausea, 14.4–14.5%; vomiting, 1.0–2.7%; osmophobia, 1.8–3.8%; photophobia, 21.2–24.5%; and phonophobia, 18.2–20.2%.

### Safety

During the double-blind phase, treatment-emergent adverse events (TEAEs) occurred in 8/132 patients (6.1%) in the safety population during almotriptan treatment and 8/132 patients (6.1%) during placebo treatment. In the mITT population (*N* = 122), there was no significant difference in the incidence of TEAEs between almotriptan and placebo (6.6% with each treatment; RR, 1.03 [95% CI, 0.40–2.66]; *p = *.9467). In the open-label phase, TEAEs were reported in 9/132 patients (6.8%). Overall, 42% of TEAEs were considered by the investigator to be definitely, possibly, or probably related to the study drug. All TEAEs were graded as mild or moderate, and none led to study discontinuation during either study phase. No individual TEAE was reported in >5% of patients, and no serious adverse events or deaths were reported during the study. No clinically relevant changes in vital signs occurred within or between treatment arms.

## Discussion

In this multicenter, double-blind, placebo-controlled, randomized, crossover study, the superiority of almotriptan over placebo for MRM attacks was demonstrated by the statistically significantly higher percentage of patients who were pain-free at two hours after medication intake. This superiority was also observed in patients with mild pain at headache onset; the study was not powered to detect a difference in patients with moderate/severe pain. In the open-label follow-up phase (two further migraine attacks), the percentage of pain-free patients at two hours with almotriptan was maintained or even increased. Results for secondary endpoints—including percentage of pain-free patients over time, sustained freedom from pain with or without adverse events, use of rescue medication and occurrence of migraine symptoms—confirmed the efficacy of almotriptan in the overall patient population. Results in the post-hoc analyses of the mild and moderate/severe subgroups also showed a benefit for almotriptan, although some results did not achieve statistical significance. It should be noted, however, that the study was not powered to detect differences between almotriptan and placebo in the post-hoc analyses.

The results of the present study are highly consistent with those from a previous retrospective analysis of almotriptan in women with MRM, in which 44.9% of patients receiving almotriptan were pain-free at two hours after medication intake (48.4% in the present study), with a recurrence rate of 32.8% (28.8% in the present study) (8). The results are also similar to those of a post-hoc analysis of the randomized, placebo-controlled AEGIS study, in which 35.4% of patients with MRM and 35.9% of those with non-MRM were pain-free at two hours post-medication, and 22.9% and 23.8%, respectively, were SPF (9).

In the present study, the safety and tolerability evaluations showed a similar profile for almotriptan and placebo, as has previously been observed both in women with MRM (8,9) and in more general migraine populations (11,12). 

The main strengths of the present study are the randomized, double-blind design and the adequate sample size for the primary analysis, although there were insufficient patients to draw definite conclusions in the post-hoc analysis of mild and moderate/severe subgroups. The patient population was sufficiently broad to allow generalization of the results to the wider population of women with MRM, with only those with very frequent or complicated (e.g. hemiplegic or basilar) migraine excluded. One possible limitation of the study is the analysis of treatment consistency, for which a more individual intrapatient assessment may have been preferred. The present results do show, however, that 56% of patients can be expected to respond to almotriptan for at least two of three migraine attacks.

In conclusion, the results of this double-blind, placebo-controlled, crossover study demonstrated the efficacy of almotriptan in ameliorating the symptoms of migraine and confirmed its superiority over placebo in women with MRM attacks, which are usually found to be of longer duration and less responsive to acute treatment than nonmenstrual attacks (13). Data from the open-label follow-up phase corroborate those of the double-blind phase and show the consistency of effect with almotriptan. Almotriptan was well tolerated throughout the study, with an incidence of adverse events similar to placebo. In light of its efficacy and tolerability profile, almotriptan can be considered a first-choice acute treatment for women with MRM, particularly if given during the mild pain phase of migraines.
